# Children’s Allocation of Study Time during the Solution of Raven’s Progressive Matrices

**DOI:** 10.3390/jintelligence6010009

**Published:** 2018-02-28

**Authors:** Patrick Perret, Bruno Dauvier

**Affiliations:** Aix Marseille Univ, PSYCLE, Aix-en-Provence, France; bruno.dauvier@univ-amu.fr

**Keywords:** reasoning, allocation of study time, cognitive development, Raven’s Progressive Matrices

## Abstract

The acuity of reasoning on Raven’s Progressive Matrices is strongly influenced by strategic determinants. Building on metamemory studies that highlight the influence of study-time allocation on memory development, we investigated children’s allocation of study time while solving these matrices. A total of 170 children aged 6–12 years completed a computerized short-form version of the standard matrices featuring items selected to represent a broad range of difficulties. Beyond analyzing changes in mean latencies and performances with age, we used generalized additive mixed models to explore within-participant variability in response times as a function of both item complexity and overall individual efficiency. Results revealed that individual differences in performances were significantly associated with children’s adaptive modulation of response times. Mediation analysis further indicated that response-time modulation contributed to age-related changes in performance. Taking account of study-time allocation in reasoning tasks may open up new avenues for the study of reasoning development and the assessment of intellectual functioning.

## 1. Introduction

Raven’s Progressive Matrices (RPM) are considered to be one of the best measures of reasoning, as attested by the ongoing presence of matrix completion tasks in most cognitive assessment batteries and by their lasting use in most research on fluid intelligence (Gf). Identifying the variables that contribute to individual differences and age-related changes in RPM performance thus offers the opportunity to broaden our understanding of reasoning development. The current study explores the hypothesis that children’s ability to adaptively modulate their study times as a function of matrix complexity constitutes a driving force for RPM performance. Our review of the literature will bring together arguments in favor of this hypothesis. Next we will present the results of an experiment in which response time’s variability was analyzed in order to reveal the adaptive modulation of study time and its relationship with age and performance. We then discuss the implications of these results with regard to theories of reasoning development and reasoning assessment practices.

When confronted with RPM items, participants have to find the missing element that completes a series of perceptually or analogically organized abstract patterns. To do so, participants have to engage in the inductive abstraction of the rules governing the organization of the successive designs. Both the nature and number of these rules contribute to the relative difficulty of the items [1]. 

The available age norms for the test clearly show that children’s reasoning abilities increase with age, but surprisingly few studies have directly addressed the question of what develops in children’s processing of matrices. Carpenter, Just and Shell [2]’s seminal study shed light on two main cognitive processes required by RPM solution: The ability to induce abstract relations and the ability to maintain and articulate sub-results. Indeed, one of the main sources of variation in RPM difficulty is the number of rules that have to be combined in order to grasp the overall structure of an item. Therefore, the solution of RPM draws heavily on working memory attentional resources and a body of empirical results supported the view that working memory development extends the scope and complexity of the items that children can solve [3,4].

Another line of hypotheses focuses on the role of processing strategies. Snow [5] identified two contrasting strategies: a constructive matching strategy that consists in mentally preparing an ideal response and then comparing it with the options that are actually available, and an elimination strategy that consists in comparing the features of the matrix elements with those of the possible responses, arriving at a default response through a process of elimination. Analyzing response times, coupled with eye movements, Vigneau, Caissie, and Bors [6] were able to confirm that individual variations in RPM efficiency are closely linked to these strategic factors, as participants with the best performances spent proportionately more time studying the matrix than they did analyzing the response options. Mitchum and Kelley [7]’s experiments also indicated that relying on a constructive matching strategy improves monitoring accuracy, with the presence of the anticipated answer among the response options providing a cue for confidence judgments. Furthermore, the processing strategies used by participants not only affect their performance, but also the validity of the test itself. When the cognitive components involved in the processing of the items differ from one strategy to another, reasoning resources are differentially engaged. For example, Arendasy and Sommer [8] showed that when participants rely on a response elimination strategy, the test’s Gf loading is reduced. Chen, Honomichl, Kennedy, and Tan [9]’s cross-sectional and microgenetic study indicated that children’s thinking becomes increasingly relational with age and experience. However, it also suggested that many children tend to underperform, insofar as minor changes in the administration procedure (e.g., verbal feedback) can help them implement more efficient strategies. This led the authors to suggest intensifying research efforts in that direction. Identifying which mechanisms facilitate the efficient processing of analogical matrices can inform the design of dynamic assessment procedures that are better able to reveal children’s core reasoning abilities [10].

The strategic dimension, therefore, seems to determine how efficiently individuals cope with the cognitive demands of RPM. However, the available literature is narrowly focused on Snow [5]’s initial distinction between constructive and elimination strategies. The present study examined another strategic dimension that might contribute to both developmental change and individual differences in performance, namely the adaptive modulation of study time. Research on human memory has consistently shown that adults regulate the study time they allocate to material as a function of both (objective) item difficulty and (subjective) judgments of learning [11]. In the absence of time pressure, items that are more difficult to memorize tend to induce longer study times, and this modulation has been found to enhance learning [12]. In the wake of Flavell’s pioneering work [13], these metacognitive processes have also been investigated from a developmental perspective. In a study by Destan, Hembacher, Ghetti, and Roebers [14], children as young as 5 years exhibited an emergent ability to differentiate between easy and difficult items, as both their judgments of learning (before the test) and their confidence judgments (after the test) varied according to the items’ complexity. However, the translation of judgments of learning into the strategic differential allocation of study times was only observed for 6- and 7-year-olds. Dufresne and Kobasigawa [15], as well as Lockl and Schneider [16], have also found similar developmental trends toward a more adaptive allocation of study time with age. In order to translate this into performances, metacognitive judgments have to be converted into effective strategic adaptations. Schneider and Pressley [17] referred to this hypothetical difficulty as an implementation deficit. Such a discrepancy between young children’s emergent abilities and their limited spontaneous use of strategies has previously been observed in other research areas, and was referred to as production deficiencies [18].

Ackerman and Thompson [19] recently proposed that these metacognitive regulatory processes, identified in research on memory, might have analogical counterparts in reasoning. As we have seen, intermediate judgments of learning help people monitor the time they allocate to memorizing material. The discrepancy reduction model [20] predicts that people will devote extra study time when they perceive the need to reduce a discrepancy between learning goals and actual judgments of learning. Ackerman and Thompson [19] suggested that, when confronted with reasoning tasks, people use similar subjective estimates of understanding to regulate the time they devote to processing information, and continue to invest efforts until a threshold of confidence is reached. Transposed to the RPM context, a first hypothesis could be that responding more slowly should promote success, as longer explorations allow for more advanced information processing. However, Vigneau, Caissie, and Bors [6]’s analyses of response times revealed a more complex pattern of data. First, speed and eye-tracking strategic indicators made distinct and complementary contributions to predicting performance. Second, the mean item latency was not related to performance. Third, only response times on easy items significantly contributed to the prediction of RPM scores. Taken together, these results led the authors to the following conclusion: “At one level, spending more time encoding critical features is an appropriate approach to solving Raven items; at another level, not spending too much time on (easy) items is a feature of efficient performance” (p. 271). More recently, Goldhammer, Naumann, and Greiff [21] examined this issue further and also found that response time effects were moderated by item difficulty. One interpretation of this result is that it is only worth devoting extra time to the processing of matrices when the item requires deeper examination, owing to the complexity of its underlying rules, and that high RPM performances are achieved through the modulation of study times according to item difficulty. In the cognitive development literature, at least one experiment suggests that children and adults may differ in the extent to which they strategically allocate different study times to different items. In a functional MRI study comparing children’s and adults’ functioning on RPM, Crone, Wendelken, van Leijenhorst, Honomichl, Christoff, and Bunge [22] incidentally observed that whereas children exhibited longer response times than adults for simple problems, this was not the case for complex ones, suggesting that children failed to allocate sufficient time to solving harder items. 

Against this background, the present study was designed to provide us with a finer-grained analysis of children’s study-time variability on RPM. More specifically, we hypothesized that an adaptive modulation of study time according to matrix complexity could contribute to individual and developmental differences in performance.

## 2. Method

Participants were 170 children (93 girls) aged 6–12 years (*M* = 9.25 years, *SD* = 1.14). They were drawn from two elementary schools located in southeastern France. Most participants were Caucasian and came from middle- to upper-class backgrounds, although data on ethnicity and socioeconomic status were not collected. Written consent to take part in this study was given by their parents, the school’s principal, and the regional supervisory school authority.

The children had to solve a computerized short-form version of the standard matrices (SRPM). We selected 24 items (6 per series B to E) to represent the various rule types and degrees of difficulty of the SRPM. The task was administered on a laptop computer (15′ screen with 1024 × 768 resolution) and the experiment was run using E-Prime 2 software (PST Inc., Sharpsburg, PA, USA).

Participants were tested individually in a quiet room provided by their school, with the experimenter seated slightly to the side of the child. Before completing the 24 test items, children were given a standard set of instructions and completed five (unselected) matrices of the SRPM as practice items. In each matrix, the bottom right-hand figure was missing, and the children were instructed to use the computer mouse to select the piece that best completed the pattern from among the response options. Accuracy and response time (RT) were registered for each item. 

## 3. Results

We began by investigating the correlations between children’s age, SRPM performances, and response times (RTs). The latter, initially expressed in milliseconds, showed a highly skewed distribution (skewness = 5.06). RTs were then log transformed, and the new variable (Log.RT) showed close to normal distribution (skewness = 0.4). The log transformation is known as an efficient way to deal with reaction times asymmetrical distributions [23] and was preferred to the inverse transformation because it does not reverse the scale, which makes it easier to read the graphs. Descriptive statistics in Table 1 shows that SRPM performances increased as a function of age, with comparable levels of between-subject variability from grade 2 to 5. The correlational analysis includes the individual mean overall Log.RT, as well as individual mean Log.RTs for easy items, intermediate items, and difficult items. Items were ranked according to the item difficulty parameter, which was one minus the success rate for the whole dataset. A modulation index was also computed as the within-participant correlation between the individual RT by item and item difficulty. A participant with short RTs for easy items and longer RTs for difficult items was thus characterized by a high positive correlation reflecting high modulation. Conversely, the absence of modulation led to an index close to zero. This individual modulation index was then correlated with the children’s age and total score on the short-form SRPM, as shown in Table 2.

**Figure 1:**
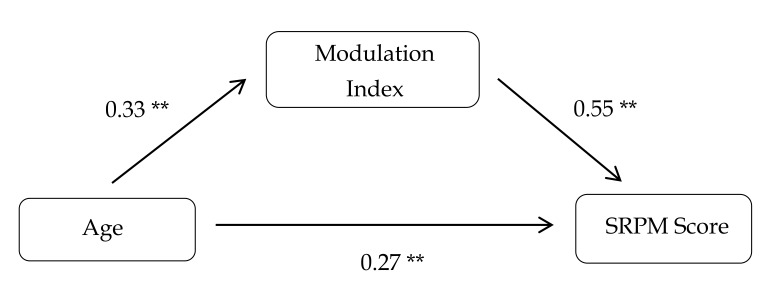
Mediation analysis of the relation between age and SRPM scores mediated by the modulation index. ** *p* < 0.01.

As expected, age and SRPM performance were correlated (*r* = 0.45, *p* < 0.01), and no correlation was found between age and overall Log.RT (*r* = 0.07, *ns*). There was a significant negative correlation between age and Log.RT for the set of easy items (*r* = −0.22, *p* < 0.01), meaning that older children spent slightly less time on these items. However, Log.RTs were positively correlated with performance, meaning that the more efficient children spent more time inspecting the items, especially the more difficult ones (*r* = 0.53, *p* < 0.01). The modulation index was positively correlated with age (*r* = 0.31, *p* < 0.01) and strongly correlated with performance (*r* = 0.63, *p* < 0.01). Children who achieved the best performances on the SRPM were also those who showed the clearest RT modulation as a function of item difficulty. Moreover, we conducted a mediation analysis to test the hypothesis that RT modulation mediated the relation between age and performance (Figure 1). The indirect effect was 0.18 (Sobel test: *z* = 4, *p* < 0.01), supporting the idea that the age-related improvement in RPM performances may be partially attributable to the development of modulation abilities.

One limitation of our modulation index is that it could only capture linear relations between item difficulty and RT at the individual level, whereas nonlinear changes could be expected if a child gave up trying to do the more difficult items. An inverted U-shaped relation would then be observed, with the child spending more time inspecting intermediate items than either easier or harder ones. To investigate the potentially nonlinear relationship between RT and item difficulty as a function of individual efficiency, we fitted a generalized additive mixed model (GAMM) to the data, using the mgcv [24] library in R [25] 1, with Log.RT as response variable, item difficulty, individual performance and the interaction between the two as explanatory variables, and an individual random intercept. GAMM models automatically identify significant nonlinear multivariate relations between variables, adopting a cross-validation approach to select the most suitable empirical degree of freedom of a nonlinear function of the predictors [27]. Random effects are also allowed within a multilevel framework [28].

**Figure 2:**
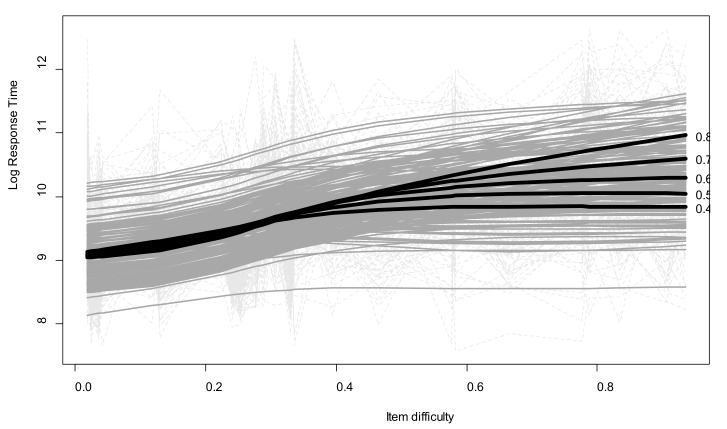
Log.RTs by item difficulty and children’s performance, with predicted values illustrating the generalized additive mixed model (GAMM) results. Light-gray dotted lines: observed values; gray lines: individual fitted values; bold black lines: predicted values for a representative subset of individuals with success rate between 0.4 and 0.8.

The model showed that the relationship between item difficulty and Log.RT was nonlinear and moderated by individual performance (interaction *edf* = 12.5, *p* < 0.01, *R*^2^ = 0.3). A graphical representation of this interaction, based on the model’s predictions for a set of representative levels of individual mean efficiency (success rate: 0.4–0.8), is provided in Figure 2. On the whole, Log.RT increased as a function of item difficulty. For the most complex items, the most efficient children spent around 11 log units (i.e., approximatively 1 min) inspecting each item, while the least efficient children spent fewer than 9.8 log units (i.e., 20 s) on each one. The increase was nearly linear for children with an SRPM success rate of around 0.8 (i.e., most efficient children), but nonlinear for children with a success rate of around 0.4. It seems that for the less efficient children in our sample, RTs increased between the easy and intermediate items, but then stayed the same for the most difficult items.

Further investigations were carried out using a relative item difficulty index that took the children’s age into account. RT modulation is supposed to be an individual mechanism that is applied as a function of item difficulty relative to the child’s own aptitude. Spending more time on a relatively difficult item than on a relatively easy item is supposed to reflect adaptive modulation, whereas spending the same amount of time on all the items corresponds to lack of modulation. Our hypothesis was that less efficient children of a given age would exhibit poorer RT regulation skills. We therefore modeled RTs in terms of (a) item difficulty relative to age, and (b) child’s aptitude compared with other children of the same age. Whereas in Figure 2 developmental and individual differences were mixed, this complementary analysis aimed at controlling for the effect of age and focusing more precisely on individual differences.

First, a Rasch model 2 was fitted to the accuracy data to obtain estimates of item difficulty and individual ability parameters on the latent continuum. This approach allowed item difficulty relative to the child’s ability to be directly estimated by subtracting the two parameters, as they were expressed on the same scale. A simple linear model was used to determine the ability level expected for each age, which then allowed us to compute the difficulty of each item relative to age. This relative item difficulty index therefore reflected how difficult an item was supposed to be at a given age, such that the higher the value, the more difficult the item. We also computed a relative child ability index reflecting the difference between a child’s expected ability given his/her age and his/her actual ability. Positive values therefore corresponded to children who performed better than expected for their age, and negative values to children who did not. A GAMM model was then fitted with Log.RT as the response variable, the relative item difficulty index, relative individual ability index and the interaction between the two as explanatory variables, and random effects allowing for a two-degree individual nonlinear trend 3. 

**Figure 3:**
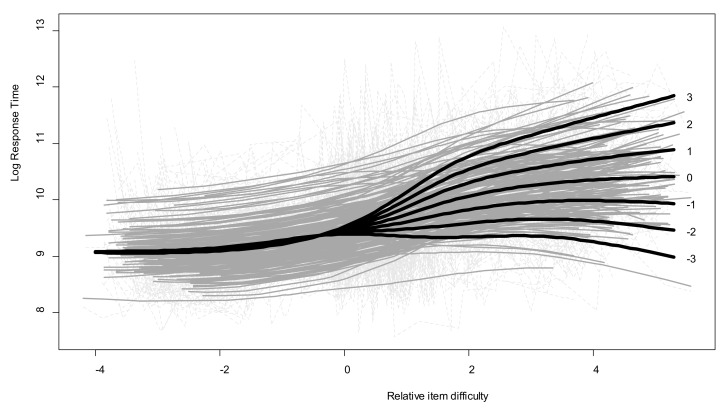
Log.RTs by item difficulty and children’s expected ability for their age, with predicted values illustrating the GAMM results. Light-gray dotted lines: observed values; gray lines: individual fitted values; bold black lines: predicted values for a representative subset of individual relative ability parameters on the Rasch scale ranging from −3 to 3.

The model showed a significant effect of the interaction between the two age-related indices on Log.RT (interaction *edf* = 10.4, *p* < 0.01, *R*^2^ = 0.26). Predicted values for a representative set of relative abilities ranging from −3 to +3 are plotted in Figure 3. One point on the age-related child ability index approximatively corresponds to one standard deviation for the group distribution (*SD* = 1.05). As the distribution was leptokurtic, extreme values around −3 and 3 were present in the sample. RT profiles only differed for difficult items. For easy items, no regulation skills were necessary, and the children gave their responses within 15 s on average (fewer than 9.5 log units). By contrast, when the difficulty of the items exceeded the ability level expected for their age, only some of the children modulated their RTs. For underachievers who performed far below expected levels (relative ability index = −3), RTs remained constant at around 10 s (9.3 log units) whatever the items’ relative difficulty. By contrast, the more difficult the items, the longer the overachievers (relative ability index = +3) spent on them, with RTs longer than 2 min (11.7 log units) for the most difficult items. Children who performed at their expected level (relative ability index = 0) exhibited a less pronounced modulation pattern, with RTs of around 30 s (10.3 log units) for the most difficult items. For these especially difficult items (relative item difficulty > 4), underachievers’ RTs were even shorter than they were for intermediate items, whereas overachievers never seemed to give up, as their RTs continuously increased as a function of item difficulty.

## 4. Discussion

In this study, we sought to examine the relationship between children’s performances on the SRPM and the time they spent studying the matrices. Drawing on memory development research, we hypothesized that children’s performances are influenced by their ability to adaptively modulate the amount of time they spend on processing matrices of different levels of difficulty. To this end, we analyzed intra-individual RT variability and computed a study-time modulation index, derived from the correlation between RT and item difficulty for each participant. Four major findings emerged from the analyses we conducted.

First, the examination of mean RTs as a function of age did not provide any insight into the mechanisms responsible for developmental change. Whereas performance showed a marked improvement with age, mean RTs did not vary significantly, suggesting an apparent (and misleading) independence of the two variables. This result clearly illustrates the need to go beyond mean-based analyses in order to further our understanding of the processes mediating developmental change and individual differences [30]. 

Second, contrary to mean RTs, the modulation index reflecting intraindividual RT variability, was significantly associated with age and performance. Older or more efficient children adjusted their study times more to the complexity of the items. Additionally, mediation analyses revealed that the age-related changes in performance were partly explained by children’s increasing modulation efficiency. Third, the relationship between RTs and item difficulty took different forms as a function of children’s level of performance. Whereas more efficient children exhibited a linear relationship between RT and difficulty (i.e., they always spent more time processing more difficult items), less efficient children tended to exhibit a nonlinear relationship. These children correctly adjusted their RTs as a function of complexity, providing the items were not too difficult, but seemed to suspend the modulation process beyond a certain threshold of difficulty. Fourth and last, when we took age-related performance expectations into account, we found evidence that underachievers and overachievers clearly displayed contrasting RT modulation profiles.

One limitation of the present study is that the strategic nature of RT modulation can still be discussed. RT modulation can be seen as a deliberate goal-oriented behavior intended to improve performance, but it could also be regarded as the byproduct of qualitatively different processing approaches, that is, as a consequence rather than a cause. For example, Dual Process Theories of reasoning [31] advocate the categorization of inferences as either produced by System 1 (intuitive and fast elicited responses) or by System 2 (analytic and time-consuming responses). These processing approaches could correspond to the recruitment of elimination and constructive matching strategies in the processing of Raven’s matrices. It could also be that RT modulation and processing approaches constitute two sides of the same coin and will be difficult to disentangle: Children cannot recruit a System 2 approach unless they accept to devote time and efforts to the processing of complex matrices, and constructive-matching strategies are themselves time-consuming. Thompson, Prowse Turner and Pennycook [32] argued that reasoning theories should integrate metacognitive components in order to address the monitoring issues that emerge from such questions: “For a given participant of a given cognitive capacity, operating under a given set of task instructions, in a given environment, what predicts the degree of Type 2 engagement?” (p. 108). With regard to the present results, this raises the straightforward research question of why some children adaptively modulate their study time while others either do not, or do so less effectively. Several hypotheses can be put forward about the nature and psychological determinants of these different profiles.

### 4.1. Role of Metacognitive Judgments

Younger or less efficient children may fail to modulate their study time because of their inability to discriminate between the items’ levels of difficulty. In the metacognitive literature, the ease of learning judgment concept refers to the estimation of difficulty that an individual can produce after brief exposure to material that has to be learned. In reasoning tasks, deliberate processing efforts and strategies may be driven by these estimations [19]. A child’s lack of study-time modulation may stem from an inability to adequately assess the difficulty of the RPM items and to discriminate between their inner complexities. Future research should try to disambiguate this issue by assessing children’s ability to produce relevant judgments of reasoning complexity.

### 4.2. Discrepancy Reduction or Region of Proximal Reasoning?

The metamemory literature that drove the hypotheses of the present study may also shed light on our results. Two main theoretical models can be used to predict children’s learning strategies. As mentioned earlier, the discrepancy reduction model predicts that children will devote more study time to items perceived as more difficult, in order to reduce the discrepancy between a desired state of learning and its actual estimation. Transposed to a reasoning context, the idea is that extra-time allocation reduces negative judgments of understanding for more complex matrices. However, Metcalfe [33] argued that a more rational learning attitude consists in concentrating resources not on more difficult items per se, but on those located in the child’s region of proximal learning, that is “those items with the smallest distance from being learned” (p. 350). The third result of our study, concerning the linear/nonlinear relationship between RTs and difficulty has several possible explanations with regard to these alternative interpretive frameworks. Should the less efficient children in Figure 2 be regarded as having a modulation deficit (i.e., not trying enough to reduce the discrepancy for the most difficult items, as the more efficient children seemed to try to do), or should they be regarded as adaptively not spending too much time and effort on the items they rapidly perceive of as being clearly out of reach? The concept of utilization deficiencies [34] describes situations in which an elaborated strategy, though available in children’s repertoire, cannot (yet) help them cope with the task at hand. Here, children may fall back to the use of simpler and faster strategic approaches when the inner complexity of an item obviously exceeds their working memory capacities, and makes the constructive-matching strategy ineffective. Future research should try to document the validity of each of these interpretations by articulating the analyses of modulation profiles with external measures of cognitive ability indicating participants’ region of proximal reasoning.

### 4.3. The Role of Thinking Dispositions

Metamemory researchers have shown that children sometimes display adequate metaknowledge, while failing to appropriately capitalize on the information it provides (e.g., [35]). The term thinking dispositions [36] refers to individual inclinations toward the effective use of available cognitive resources. The motivational aspect of research on intelligence indicates that cognitive sophistication results from both processing abilities and thinking dispositions (e.g., [37]), such that skills are not enough: “However technically adroit a person may be at problem solving, decision making, reasoning, or building explanations, what does it matter unless the person invests himself or herself energetically in these and other kinds of thinking on occasions that invite it?” [36] (p. 276). In order to solve the SRPM, children have to engage in a reiterated and effortful process of induction that draws heavily on (limited) relational integration abilities in working memory. In the test manual, Raven, Raven, and Court [38] highlighted this dispositional dimension, claiming that one of the main sources of error is the reluctance to devote mental energy to solving abstract problems. Vodegel-Matzen, van der Molen, and Dudink [1] found that high-scoring individuals typically spent more time solving the items than low scorers. In a similar vein, Kagan, Pearson, and Welch [39] had earlier highlighted the influence of cognitive style (impulsive vs. reflective) on reasoning test performances. The fourth result of our study indicated that age-related expectancies of success/failure on a given item could be contradicted by actual performances as a function of the time (and certainly the effort) a child devotes to exploring the matrix. As shown in Figure 3, underachievers tended to invest equivalent amounts of time in processing the items, whatever their attainability. Overachievers showed the opposite pattern, persevering proportionally to the magnitude of the challenge the items represented for them. This last result points to the need to consider thinking dispositions (e.g., effort willingness, need for cognition, cognitive style) in future research exploring modulation profiles.

Overall, the findings of the present study provide evidence that study-time modulation constitutes one of the key strategic factors for developmental and individual differences in reasoning performances. Additional research is now needed to further explore the determinants of children’s modulation profiles and to gain a deeper understanding of their actual contribution to intelligent behaviors. On the clinical front, which is moving toward the digitization of cognitive assessment procedures, taking account of study-time modulation during the solution of matrix reasoning tasks could greatly enrich analysis of the processes behind the scores. These analyses could guide both clinical assessment and cognitive remediation: Children displaying obviously dysfunctional RT modulation, such as the underachievers in Figure 3, could benefit from dynamic assessment procedures designed to increase the validity of Gf measures [40].

## Figures and Tables

**Table 1:** Descriptive statistics mean (standard deviation) of age, short-form version of the standard matrices (SRPM) performances, response times (RTs) by grade group.

Grades	2	3	4	5
N	46	38	43	43
Age	95.3 (5.04)	105.6 (3.7)	117.3 (4.38)	129.3 (4.09)
SRPM perf.	0.49 (0.17)	0.6 (0.13)	0.63 (0.13)	0.7 (0.1)
Log.RT	9.76 (0.88)	9.71 (0.84)	9.83 (0.93)	9.58 (0.86)

**Table 2:** Descriptive statistics and correlations between age, SRPM performances, RTs and the modulation index.

	Mean (SD)	Modul.	Perf.	Log.RT	Log.RT-e	Log.RT-i	Log.RT-d
Age (months)	111.8 (13.71)	0.31 **	0.45 **	−0.07	−0.22 **	−0.14	0.12
Modulation	0.52 (0.24)	—	0.63 **	0.15 *	−0.27 **	−0.03	0.56 **
SRPM perf.	0.6 (0.15)	0.63 **	—	0.24 **	−0.1	0.07	0.53 **

Note: SRPM perf.: individual success rate; Log.RT-e (-i, -d): individual mean Log.RT for easy (-e), intermediate (-i), or difficult (-d) items. * *p* < 0.05. ** *p* < 0.01.

## Footnotes

1 The mgcv library in R was used to fit the binomial GAMM model with the tensor (te) function to test for nonlinear interactions. The model formula, expressed in Wilkinson and Rogers ‘s notation [26], was LogIT~te(ItemDifficulty,IndividualPerformance) + (1|Subject).

2 The Rasch model was fitted using the eMr package [29] in R, which allows for maximum likelihood estimation of individual parameters.

3 LogTI~te(RelativeItemDifficulty, RelativeChildAbility) + (ns(RelativeItemDifficulty, 2)|Subject). The ns (…, 2) function gives the natural splines with two degrees of freedom, and allows for nonlinear random effects.

## References

[1] Vodegel Matzen L.B., van der Molen M.W., Dudink A.C. (1994). Error analysis of Raven test performance. Personal. Individ. Differ..

[2] Carpenter P.A., Just M.A., Shell P. (1990). What one intelligence test measures: A theoretical account of the processing in the Raven Progressive Matrices test. Psychol. Rev..

[3] Pennings A.B., Hessels M.G.P. (1996). The measurement of mental attentional capacity: A neo-Piagetian developmental study. Intelligence.

[4] Dauvier B., Bailleux C., Perret P. (2014). The development of relational integration during childhood. Dev. Psychol..

[5] Snow R.E., Snow R.E., Federico P.-A., Montague W.E. (1980). Aptitude processes. Aptitude, Learning, and Instruction: Cognitive Process Analyses of Aptitude.

[6] Vigneau F., Caissie A.F., Bors D.A. (2006). Eye-movement analysis demonstrates strategic influences on intelligence. Intelligence.

[7] Mitchum A.L., Kelley C.M. (2010). Solve the problem first: Constructive solution strategies can influence the accuracy of retrospective confidence judgments. J. Exp. Psychol. Learn. Memory Cognit..

[8] Arendasy M.E., Sommer M. (2013). Reducing response elimination strategies enhances the construct validity of figural matrices. Intelligence.

[9] Chen Z., Honomichl R., Kennedy D., Tan E. (2016). Aiming to complete the matrix: Eye-movement analysis of processing strategies in children’s relational thinking. Dev. Psychology.

[10] Resing W.C.M., Bakker M., Pronk C.M.E., Elliott J.G. (2017). Progression paths in children’s problem solving: The influence of dynamic testing, initial variability, and working memory. J. Exp. Child Psychol..

[11] Son L.K., Metcalfe J. (2000). Metacognitive and control strategies in study-time allocation. J. Exp. Psychol. Learn. Memory Cognit..

[12] Metcalfe J. (2009). Metacognitive judgments and control of study. Curr. Dir. Psychol. Sci..

[13] Flavell J.H., Wellman H.M., Kail R.V., Hagen J.W. (1977). Metamemory. Perspectives on the Development of Memory and Cognition.

[14] Destan N., Hembacher E., Ghetti S., Roebers C. (2014). Early metacognitive abilities: The interplay of monitoring and control processes in 5- to 7-year-old children. J. Exp. Child Psychol..

[15] Dufresne A., Kobasigawa A. (1989). Children’s spontaneous allocation of study time: Differential and sufficient aspects. J. Exp. Child Psychol..

[16] Lockl K., Schneider W. (2004). The effects of incentives and instructions on children’s allocation of study time. Eur. J. Dev. Psychol..

[17] Schneider W., Pressley M. (1997). Memory Development between Two and Twenty.

[18] Bjorklund D.F., Miller P.H. (1997). New themes in strategy development. Dev. Rev..

[19] Ackerman R., Thompson V.A., Feeney A., Thompson V.A. (2014). Meta-reasoning: What can we learn from metamemory. Reasoning as Memory.

[20] Nelson T.O., Narens L., Bower G.H. (1990). Metamemory: A theoretical framework and new findings. The Psychology of Learning and Motivation.

[21] Goldhammer F., Naumann J., Greiff S. (2015). More is not always better: The relation between item response and item response time in Raven’s Matrices. J. Intell..

[22] Crone E.A., Wendelken C., van Leijenhorst L., Honomichl R.D., Christoff K., Bunge S.A. (2009). Neurocognitive development of relational reasoning. Dev. Sci..

[23] Ratcliff R. (1993). Methods for dealing with reaction time outliers. Psychol. Bull..

[24] Wood S.N. (2011). Fast stable restricted maximum likelihood and marginal likelihood estimation of semiparametric generalized linear models. J. R. Stat. Soc..

[25] R Core Team (2017). R: A Language and Environment for Statistical Computing.

[26] Wilkinson G., Rogers C. (1973). Symbolic description of factorial models for the analysis of variance. Appl. Stat..

[27] Wood S.N. (2004). Stable and efficient multiple smoothing parameter estimation for generalized additive models. J. Am. Stat. Assoc..

[28] Wood S.N. (2017). Generalized Additive Models: An Introduction with R.

[29] Mair P., Hatzinger R. (2007). Extended Rasch modeling: The eRm package, for the application of IRT models in R. J. Stat. Softw..

[30] Mella N., Fagot D., Lecerf T., de Ribaupierre A. (2015). Working memory and intraindividual variability in processing speed: A lifespan developmental and individual differences study. Mem. Cognit..

[31] Evans J.S.B.T. (2006). The heuristic-analytic theory of reasoning: Extension and evaluation. Psychon. Bull. Rev..

[32] Thompson V.A., Prowse Turner J.A., Pennycook G. (2011). Intuition, reason, and metacognition. Cognit. Psychol..

[33] Metcalfe J. (2002). Is study time allocated selectively to a region of proximal learning?. J. Exp. Psychol. Gen..

[34] Miller P.H. (1994). Individual differences in children’s strategic behaviors: Utilization deficiencies. Learn. Individ. Differ..

[35] Metcalfe J., Finn B. (2013). Metacognition and control of study choice in children. Metacognit. Learn..

[36] Perkins D. (1995). Outsmarting IQ: The Emerging Science of Learnable Intelligence.

[37] Toplak M.E., West R.F., Stanovich K.E. (2014). Rational thinking and cognitive sophistication: Development, cognitive abilities, and thinking dispositions. Dev. Psychol..

[38] Raven J., Raven J.C., Court J.H. (1998). Manual for Raven’s Progressive Matrices and Vocabulary Scales. Section 1: General Overview.

[39] Kagan J., Pearson L., Welch L. (1966). Conceptual impulsivity and inductive reasoning. Child Dev..

[40] Elliott J. (2003). Dynamic assessment in educational settings: Realizing potential. Educ. Rev..

